# Supplementary material for: Induced Arp2/3 Complex Depletion Increases FMNL2/3 Formin Expression and Filopodia Formation
Source: Front Cell Dev Biol. 2021 Feb 1;9:634708. doi: 10.3389/fcell.2021.634708 (PMC7882613; doi:10.3389/fcell.2021.634708)
Supplement: Supplementary file 7 [file Data_Sheet_1.PDF]

Supplementary Material

Supplementary Figures

Figure S1.

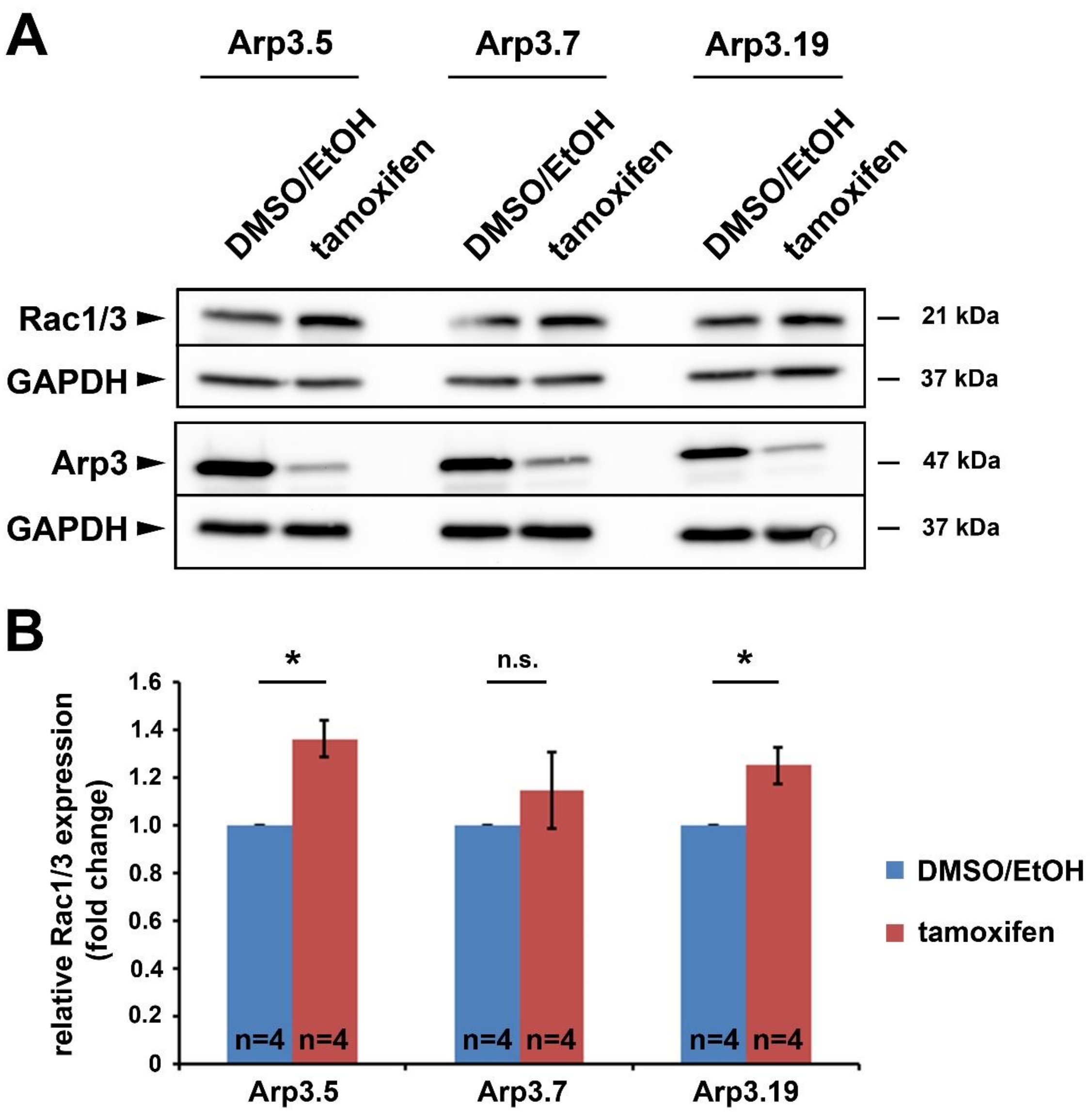

Arp3 depletion increases Rac expression levels. (A) Representative Western blot showing Rac protein levels using an antibody cross-reactive with Rac1 and -3 on cell extracts from *Actr3*$^{fl/fl}$ cells

with and without Tamoxifen treatment. GAPDH served as loading control. **(B)** Quantification of Rac protein levels from Western blots as described in (A). Bar charts display arithmetic means of Rac protein normalized to protein levels of GAPDH, with each knockout cell population (red) being presented as fold-change to its respective control (blue). Error bars represent standard error of means. Data were obtained from four independent experiments. Statistics were performed using non-parametric, Mann-Whitney rank sum test. Increase of Rac levels upon Arp3 removal were confirmed to be statistically significant in Arp3.5 and Arp3.19 cells (* p<0.05), and at least followed the same trend in clone Arp3.7 (n.s. not significant).

Figure S2.

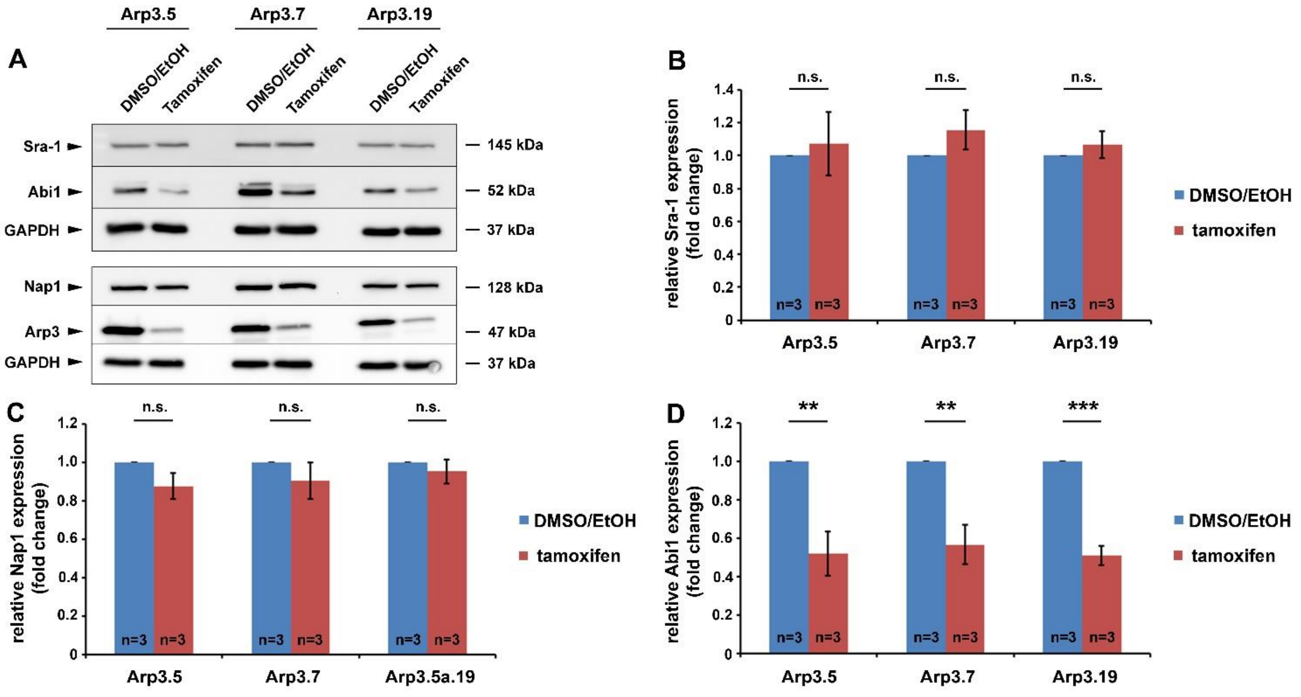

Expression levels of the WRC subunit Abi1 are decreased upon acute Arp3 knockout. (A) Representative Western blot for WRC subunits Sra-1, Abi1 and Nap1 in *Actr3*$^{fl/fl}$ clones with and without tamoxifen treatment, as indicated. For Nap1 expression, lysates used were identical to the ones used for Rac protein quantification in Fig. S1, hence Arp3 and GAPDH bands are identical. GAPDH served as loading control. **(B/C/D)** Quantification of protein levels from Western blots as shown in (A). Bar charts display arithmetic means +/- SEMs for Sra-1 **(B)**, Nap1 **(C)** and Abi1 **(D)** protein levels, as indicated, and normalized to GAPDH, with each knockout (red) being presented as fold-change to its respective control (blue). Data were obtained from three independent experiments, and statistics performed using non-parametric, Mann-Whitney rank sum test, confirming differences for Abi1 protein levels to be statistically significant (* p<0.05, *** p<0.001, n.s. not significant).

Figure S3.

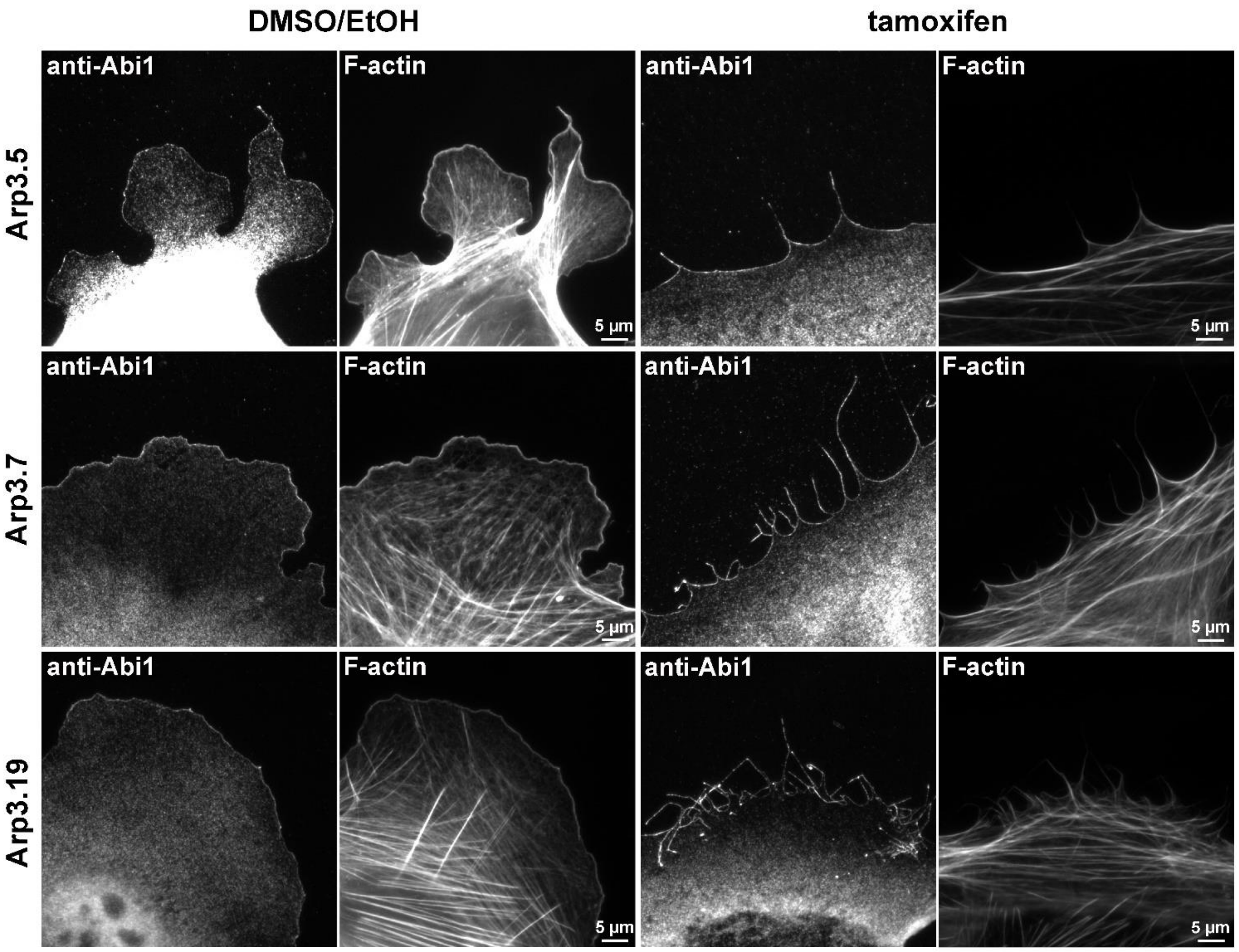

Loss of lamellipodia upon Arp3 removal does not coincide with displacement of Abi from the cell periphery. Immunolabeling with anti-Abi1 antibodies and counterstaining for the actin cytoskeleton of *Actr3*$^{fl/fl}$ clones with or without tamoxifen treatment, as indicated. Note the association of the WRC subunit Abi1 with peripheral F-actin bundles despite the absence of lamellipodia in tamoxifen-treated cells, implying that lamellipodia cannot form due to the absence of Arp2/3 complex, and not WRC.

Figure S4.

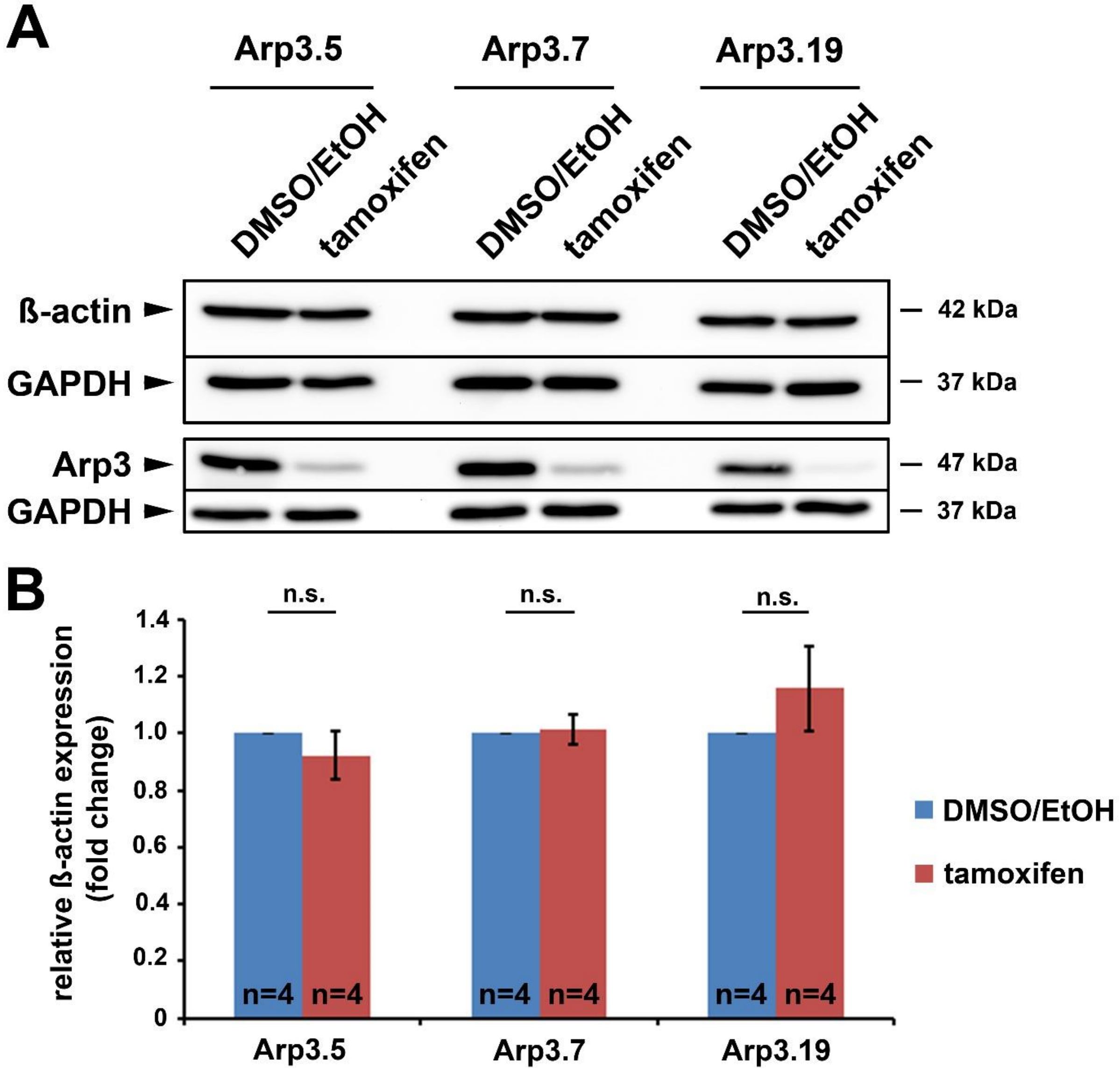

Acute Arp3 knockout does not alter ß-actin levels. (A) Representative Western blot of ß-actin levels in *Arp3*$^{fl/fl}$ clones with or without tamoxifen treatment, as indicated. GAPDH served as loading control. Note efficient Arp3 protein rundown after tamoxifen treatment. **(B)** Quantification of ß-actin levels form Western blots as shown in (A). Bar charts are arithmetic means +/- SEMs of ß-actin levels normalized to GAPDH, with each knockout (red) being presented as fold-change to its respective control (blue). Data were obtained from four independent experiments, and statistics done using non-parametric, Mann-Whitney rank sum test (n.s. not significant).

Figure S5.

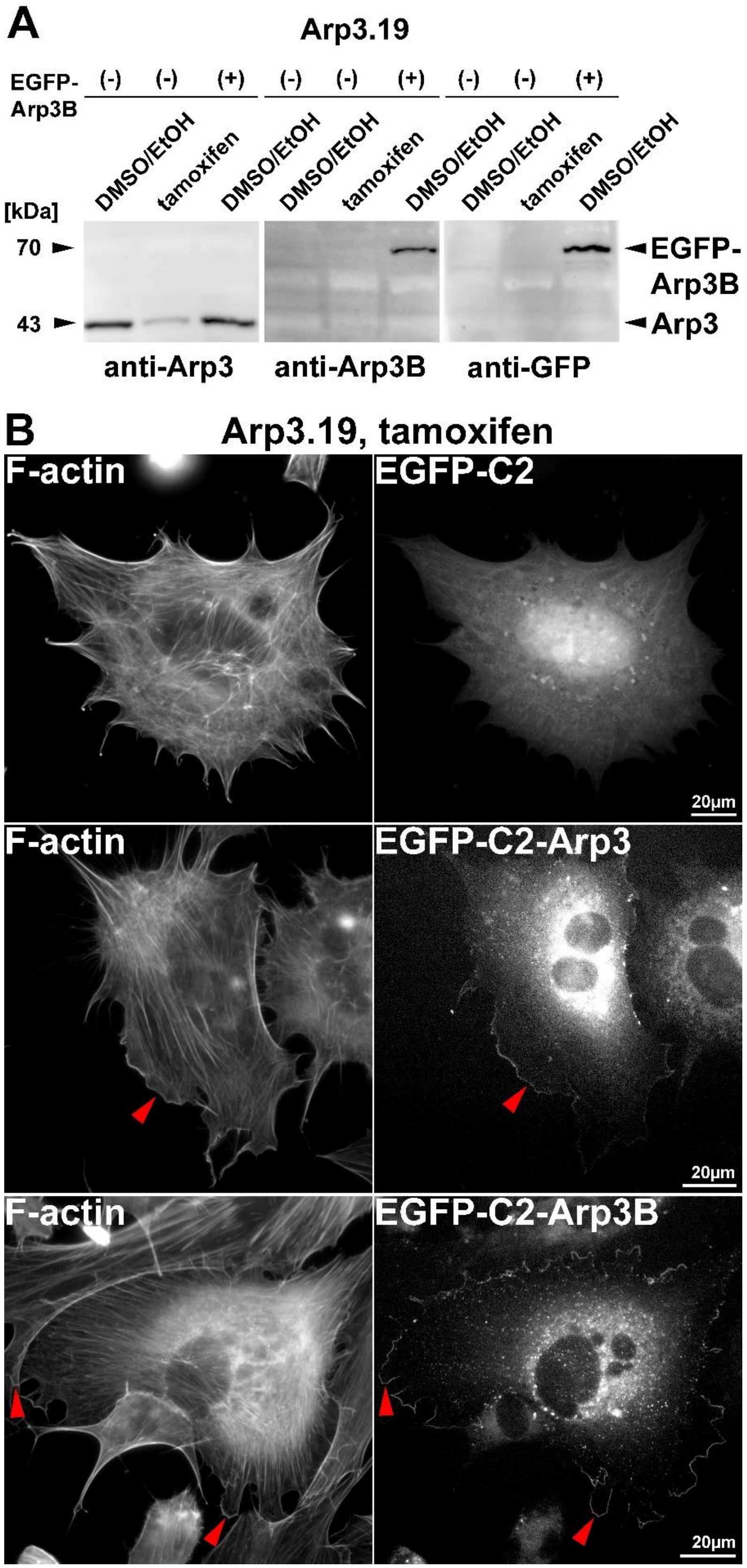
A
Arp3.19
EGFP-Arp3B
(-) (-) (+) (-) (-) (+) (-) (-) (+)
DMSO/EtOH
tamoxifen
DMSO/EtOH
DMSO/EtOH
tamoxifen
DMSO/EtOH
DMSO/EtOH
tamoxifen
DMSO/EtOH
[kDa]
70
43
EGFP-Arp3B
Arp3
anti-Arp3
anti-Arp3B
anti-GFP
B
Arp3.19, tamoxifen
F-actin
EGFP-C2
20µm
F-actin
EGFP-C2-Arp3
20µm
F-actin
EGFP-C2-Arp3B
20µm

Overexpression of both Arp3 and Arp3B restores lamellipodia formation upon removal of endogenous Arp3. (A) Representative Western blot analysis of Arp3 and Arp3B expression in *Actr3*$^{fl/fl}$ cells (clone Arp3.19) in control conditions (DMSO/EtOH) or after induction of *Actr3* knockout (tamoxifen). Arp3-specific antibody confirmed endogenous expression of Arp3 in DMSO/EtOH-treated cells as well as its successful depletion upon tamoxifen treatment. In contrast, anti-Arp3B antibody is specific for Arp3B protein, as illustrated by overexpression of EGFP-tagged Arp3B, but does not appear to be expressed (with or without Arp3 removal) from its endogenous locus (*Actr3b*). Anti-GFP antibodies served as control for EGFP-Arp3B transfection, as indicated. **(B)** Phalloidin stainings (left panels) of Arp3.19 cells following tamoxifen treatment and transient transfection with either EGFP-C2-blank, EGFP-C2-Arp3 or EGFP-C2-Arp3B (right panels), the two latter of which targeted to the cell periphery, as expected (red arrowheads). Note the restoration of lamellipodia (red arrowheads) with both EGFP-tagged Arp3 and Arp3B, but not EGFP alone, strongly suggesting the absence of expression of functional protein levels from the endogenous *Actr3b* gene upon *Actr3* disruption.

Figure S6.

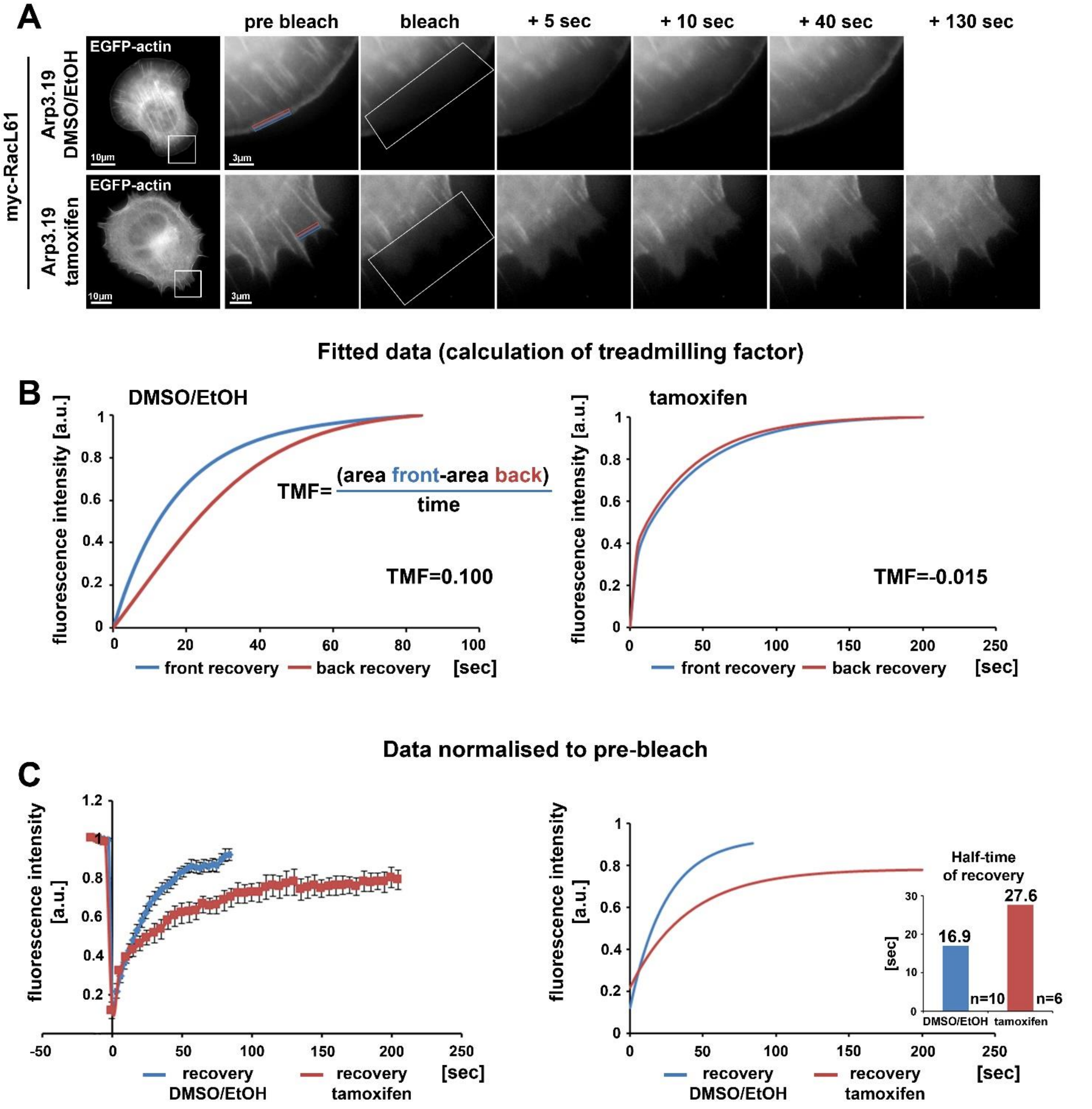

Loss of the Arp2/3 complex interferes with treadmilling and F-actin turnover even upon expression of constitutively active Rac. Experiments and analyses shown were as described for Figure 3, except that clone Arp3.19 cells were co-transfected with EGFP-actin and constitutively active Rac1. Movies from 10 control and 6 tamoxifen-treated cells acquired on three independent experimental days were analyzed.

Figure S7.

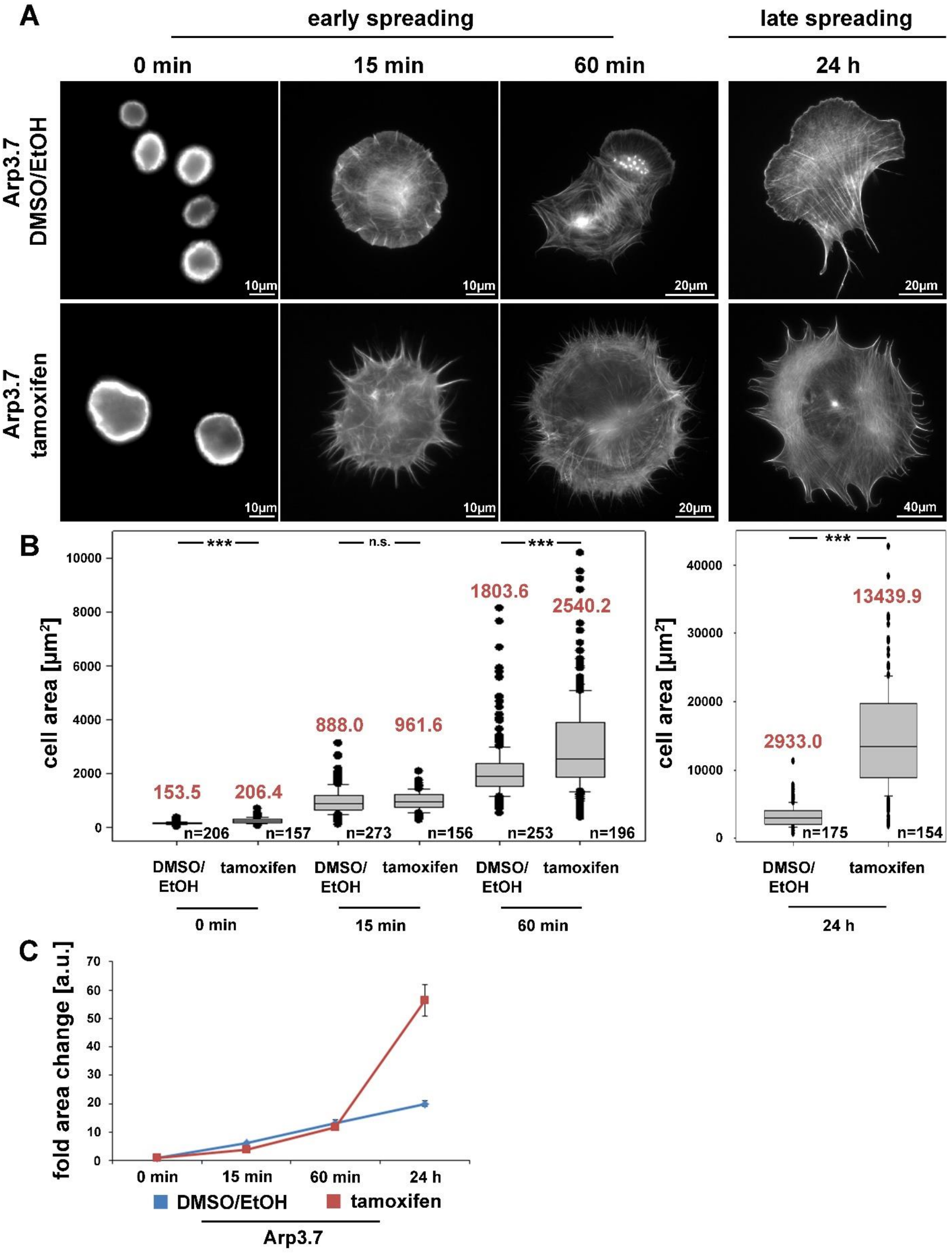
A
early spreading
late spreading
0 min
15 min
60 min
24 h
Arp3.7
DMSO/EtOH
Arp3.7
tamoxifen
10µm
10µm
20µm
20µm
10µm
10µm
20µm
40µm
B
***
n.s.
***
***
cell area [µm²]
153.5
206.4
888.0
961.6
1803.6
2540.2
2933.0
13439.9
n=206
n=157
n=273
n=156
n=253
n=196
n=175
n=154
DMSO/
EtOH
tamoxifen
0 min
15 min
60 min
24 h
C
fold area change [a.u.]
0 min
15 min
60 min
24 h
DMSO/EtOH
tamoxifen
Arp3.7

The Arp2/3 complex is not required for cell spreading, clone Arp3.7. Data generated and analyzed in analogy to those shown for clone Arp3.19 in Fig. 4.

Figure S8.

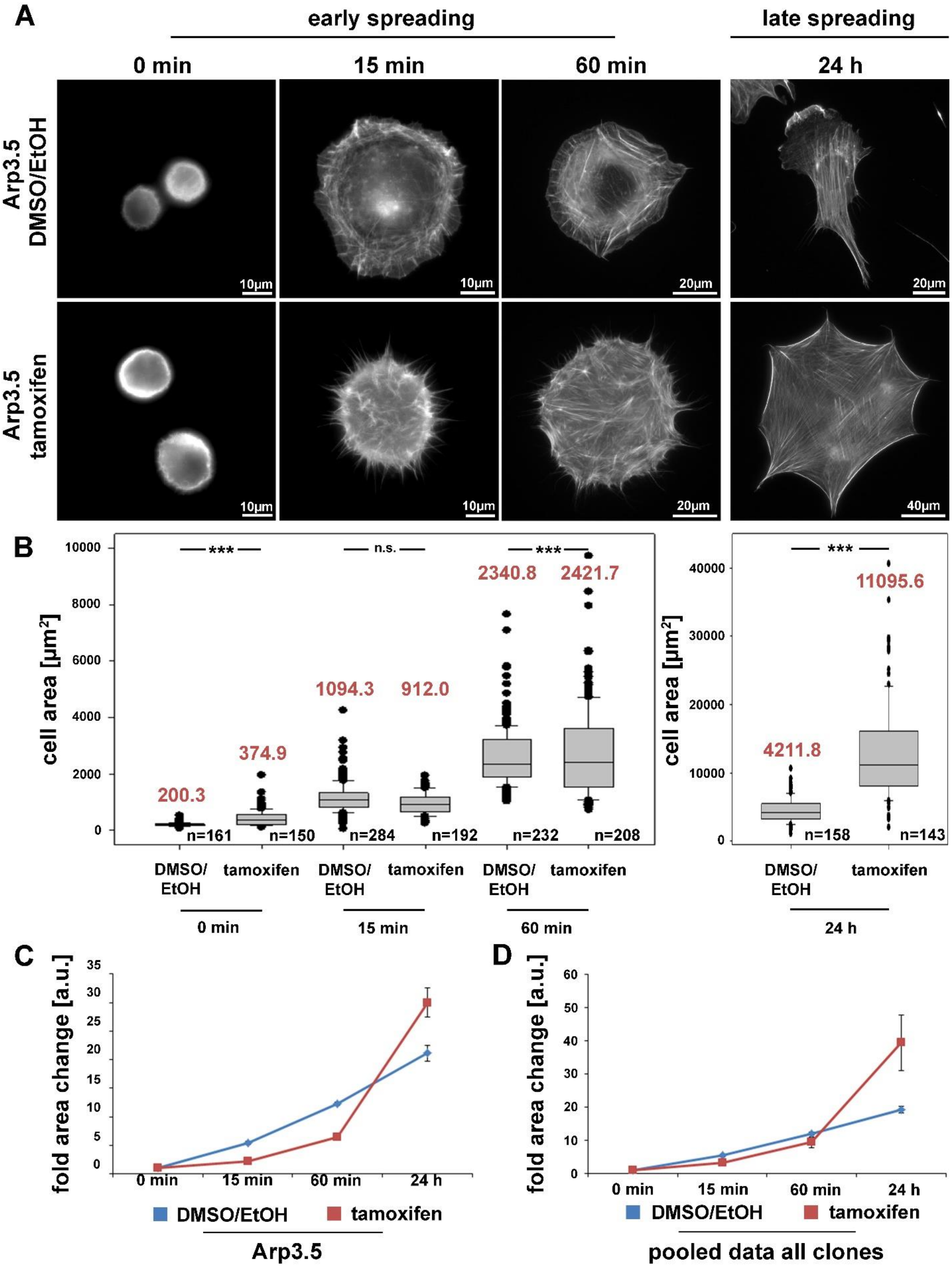
A
early spreading
late spreading
0 min
15 min
60 min
24 h
Arp3.5 DMSO/EtOH
Arp3.5 tamoxifen
10µm
20µm
40µm
B
cell area [µm2]
***
n.s.
200.3
374.9
1094.3
912.0
2340.8
2421.7
4211.8
11095.6
n=161
n=150
n=284
n=192
n=232
n=208
n=158
n=143
DMSO/ EtOH
tamoxifen
0 min
15 min
60 min
24 h
C
fold area change [a.u.]
0 min
15 min
60 min
24 h
DMSO/EtOH
tamoxifen
Arp3.5
D
fold area change [a.u.]
DMSO/EtOH
tamoxifen
pooled data all clones

The Arp2/3 complex is not required for cell spreading, clone Arp3.5. Data generated and analyzed in analogy to those shown for clone Arp3.19 in Fig. 4. In (C), cells corresponding to clone Arp3.5 display a slight delay in spreading efficiency if normalized to the strong differences in cell size at the onset of spreading (0 min-time point), which was interpreted as clonal variation as opposed to genotype-dependent difference, because not seen for clones Arp3.19 (Fig. 4) and Arp3.7 (Fig. S7) treated with tamoxifen. This view is confirmed when pooling data from all three clones, as shown in **(D)**.

Figure S9.

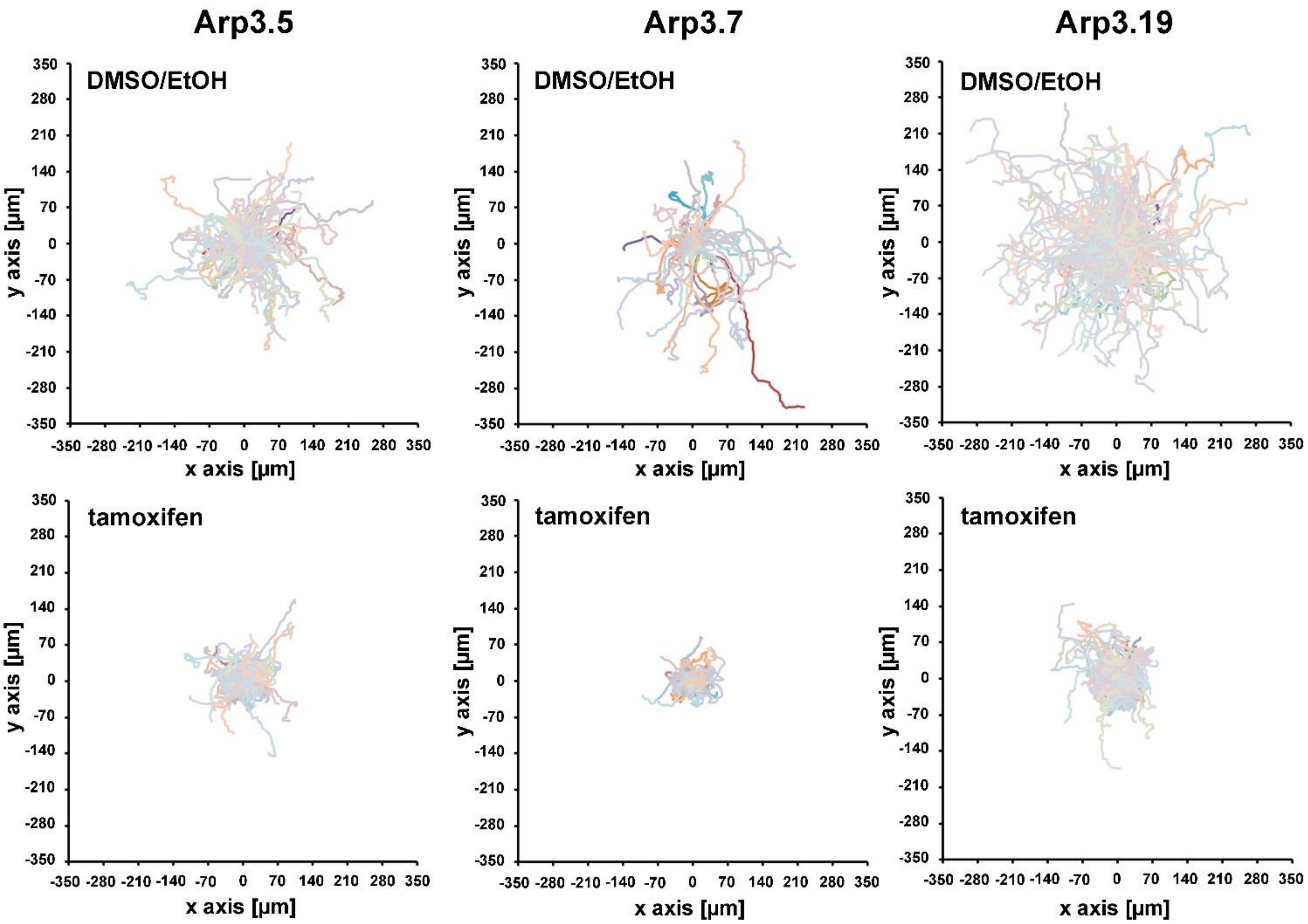

Defects of random cell migration efficiency in spite of nearly unchanged speeds. Individual cell tracks from random cell migration data for all cell clones and control (DMSO/EtOH) *versus* tamoxifen-treated conditions, as indicated. Data illustrate the tracks obtained from the analysis corresponding to the results shown in Figure 6.

Figure S10.

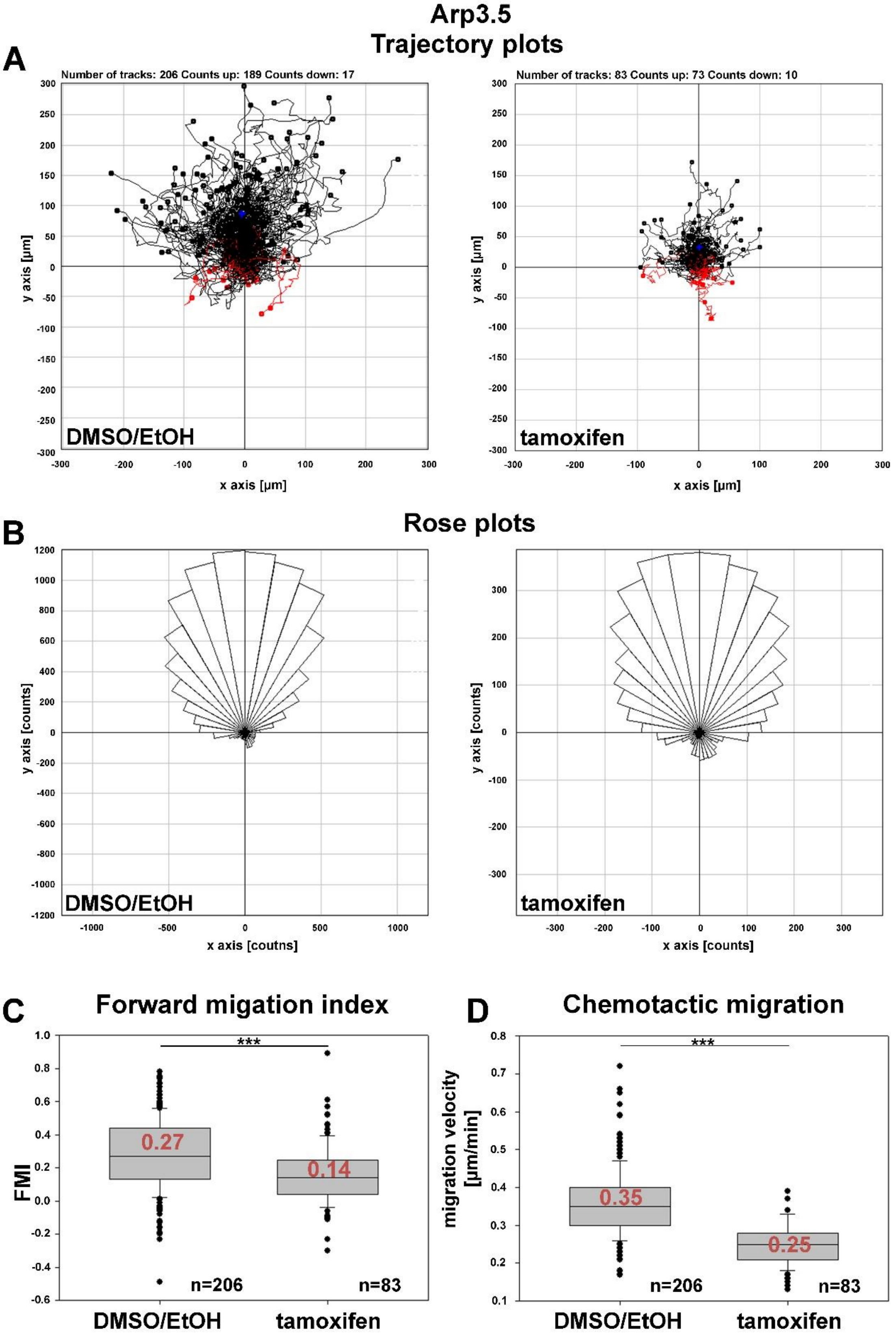

Arp3.5
Trajectory plots
A
Number of tracks: 206 Counts up: 189 Counts down: 17
Number of tracks: 83 Counts up: 73 Counts down: 10
y axis [µm]
x axis [µm]
DMSO/EtOH
tamoxifen
B
Rose plots
y axis [counts]
x axis [coutns]
x axis [counts]
DMSO/EtOH
tamoxifen
C
Forward migation index
FMI
0.27
0.14
***
n=206
n=83
DMSO/EtOH
tamoxifen
D
Chemotactic migration
migration velocity [µm/min]
0.35
0.25
***
n=206
n=83
DMSO/EtOH
tamoxifen

Arp2/3 complex contributes to, but is not essential for chemotaxis, clone Arp3.5. Data generated and analyzed in analogy to those shown for clone Arp3.19 in Fig. 7.

Figure S11.

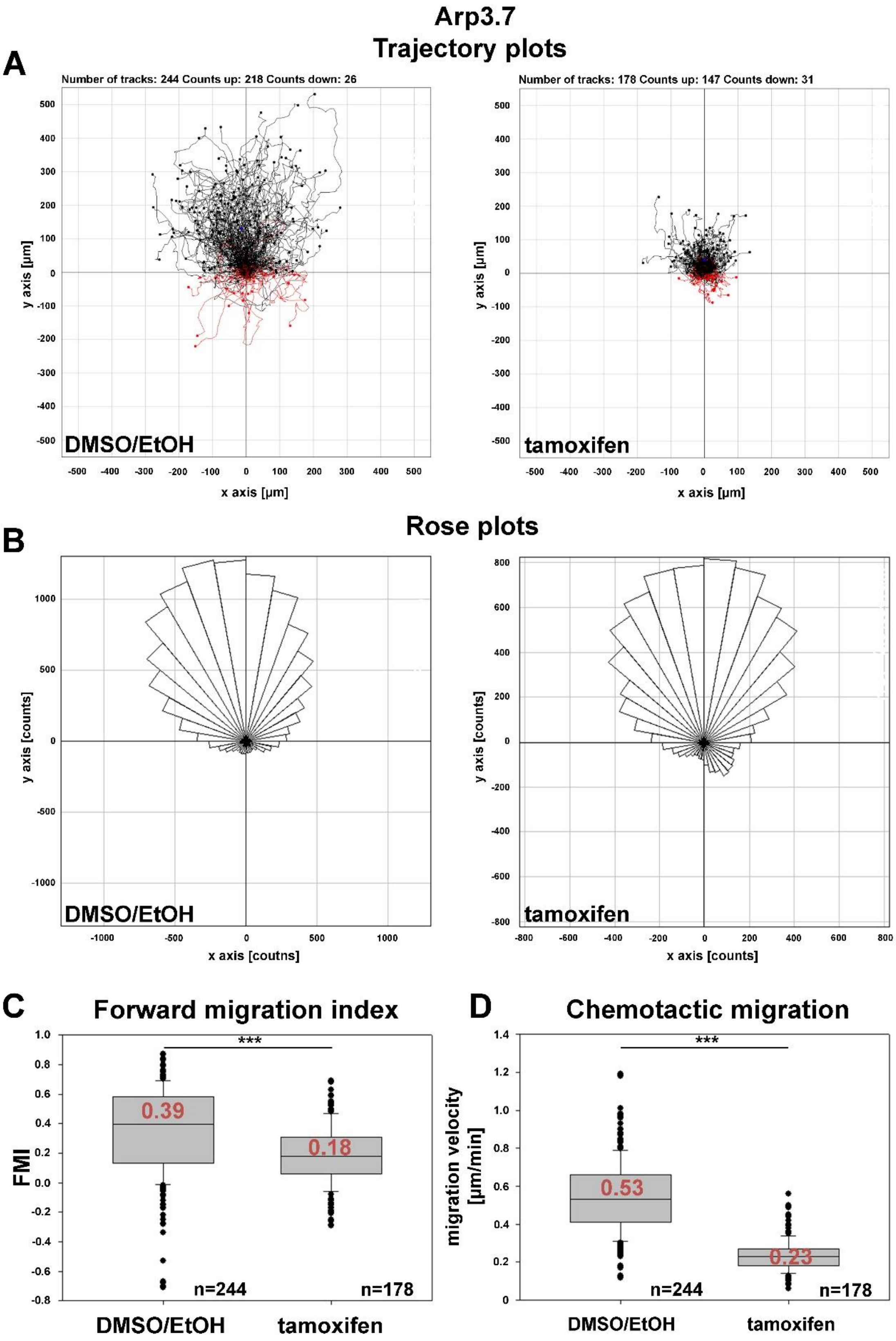

Arp3.7
Trajectory plots
A
Number of tracks: 244 Counts up: 218 Counts down: 26
Number of tracks: 178 Counts up: 147 Counts down: 31
y axis [µm]
x axis [µm]
DMSO/EtOH
tamoxifen
B
Rose plots
y axis [counts]
x axis [coutns]
x axis [counts]
DMSO/EtOH
tamoxifen
C
Forward migration index
FMI
0.39
0.18
***
n=244
n=178
DMSO/EtOH
tamoxifen
D
Chemotactic migration
migration velocity [µm/min]
0.53
0.23
***
n=244
n=178
DMSO/EtOH
tamoxifen

Arp2/3 complex contributes to, but is not essential for chemotaxis, clone Arp3.7. Data generated and analyzed in analogy to those shown for clone Arp3.19 in Fig. 7.

Figure S12.

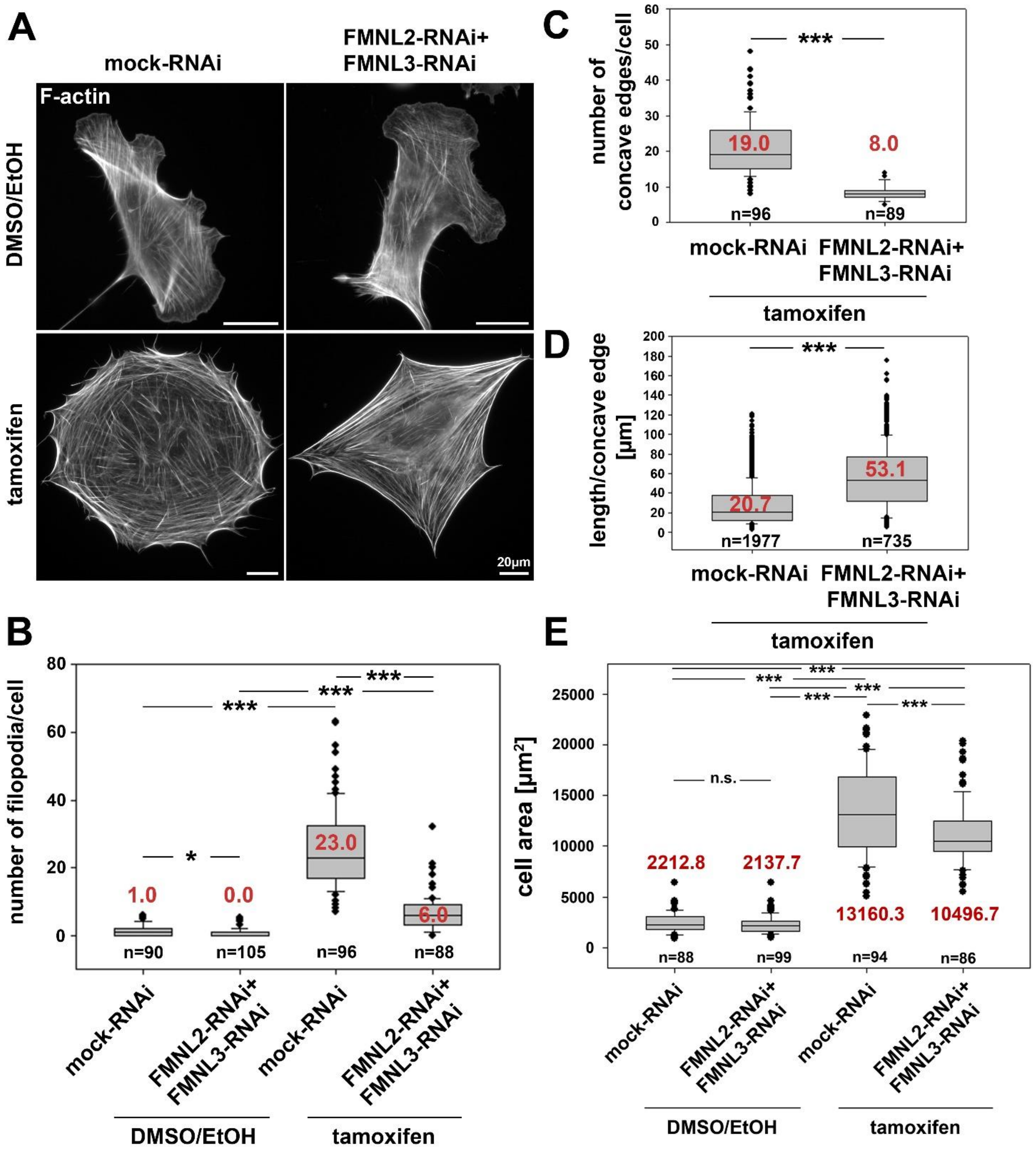

Depletion of FMNL formins reduces cell edge complexity in Arp3 knockout fibroblasts. (A) Representative phalloidin stainings of spread, DMSO/EtOH- and tamoxifen-treated *Actr3*$^{fl/fl}$ MEFs (clone Arp3.19) subjected to either mock-RNAi or FMNL2+3-RNAi. **(B)** Quantification of filopodia numbers/cell in conditions as depicted in (A). Data are presented as box and whiskers plots as described for Fig. 4B. n equals total number of cells analyzed from three independent experiments. Differences in filopodia numbers/cell between experimental conditions were confirmed to be statistically significant using non-parametric, Mann-Whitney rank sum test (* $p<0.05$; *** $p<0.001$). Note the induction of filopodia formation by acute Arp3 removal even in readily spread cells, which appears largely effected by elevated FMNL protein levels, as quantified in Fig. 9B. **(C/D)** Box and whiskers plots displaying manual quantification of the number of concave edges defining the cell periphery of tamoxifen-treated *Arp3*$^{fl/fl}$ cells (C) and length measurements of those concave edges (D) using images as displayed in (A). n corresponds to the total number of cells analyzed (C) and total number of measured concave edges (D) from three independent experiments. Statistics was done using non-parametric, Mann-Whitney rank sum test (*** $p<0.001$). Note that upon depletion of Arp3, the cell edge is not only characterized by an increase in filopodia numbers, but also by the formation of many concave edges of short length (A). However, the number of these concave edges is significantly reduced with the length of each concave edge being significantly increased once Arp3 removal is combined with RNAi-mediated suppression of FMNL2/3 formin expression. As a result, cells reduced for both Arp3 and FMNL2/3 expression display less complex cell edges (C) with concave edges being increased in length (D) as compared to cells lacking Arp3 alone. **(E)** Quantification of cell area in conditions as depicted in (A), and data presented as described for (B). Differences in cell area between experimental conditions were confirmed to be statistically significant using non-parametric, Mann-Whitney rank sum test (n.s. not significant; *** $p<0.001$).

Figure S13.

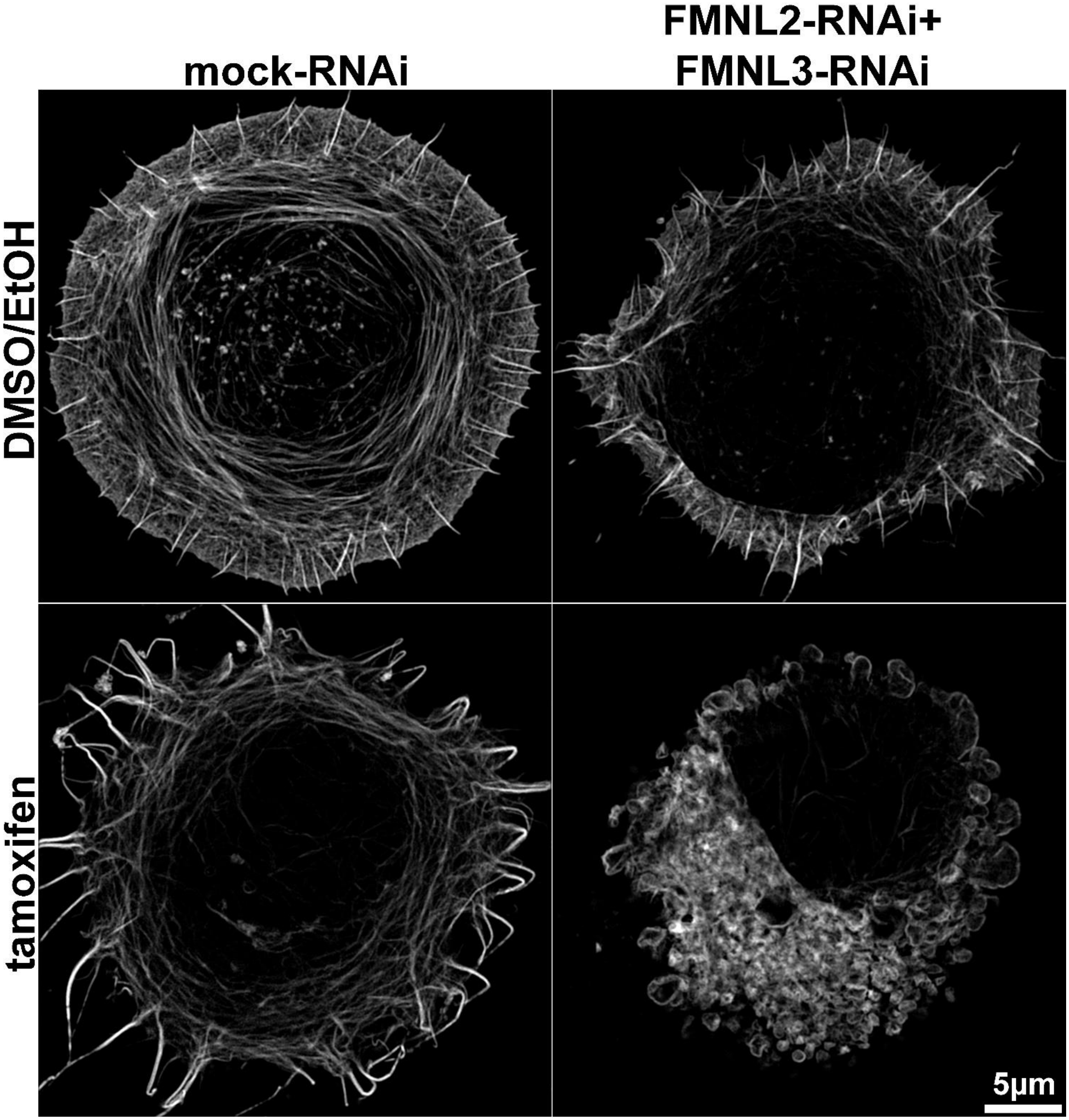

Morphological consequences of individual and combined interference with Arp3 and FMNL2/3 formin function. Structured illumination microscopy (SIM) images of phalloidin-stained cells allowed to spread for 15 minutes following genetic as well as combined RNAi treatments, as indicated.

Figure S14.

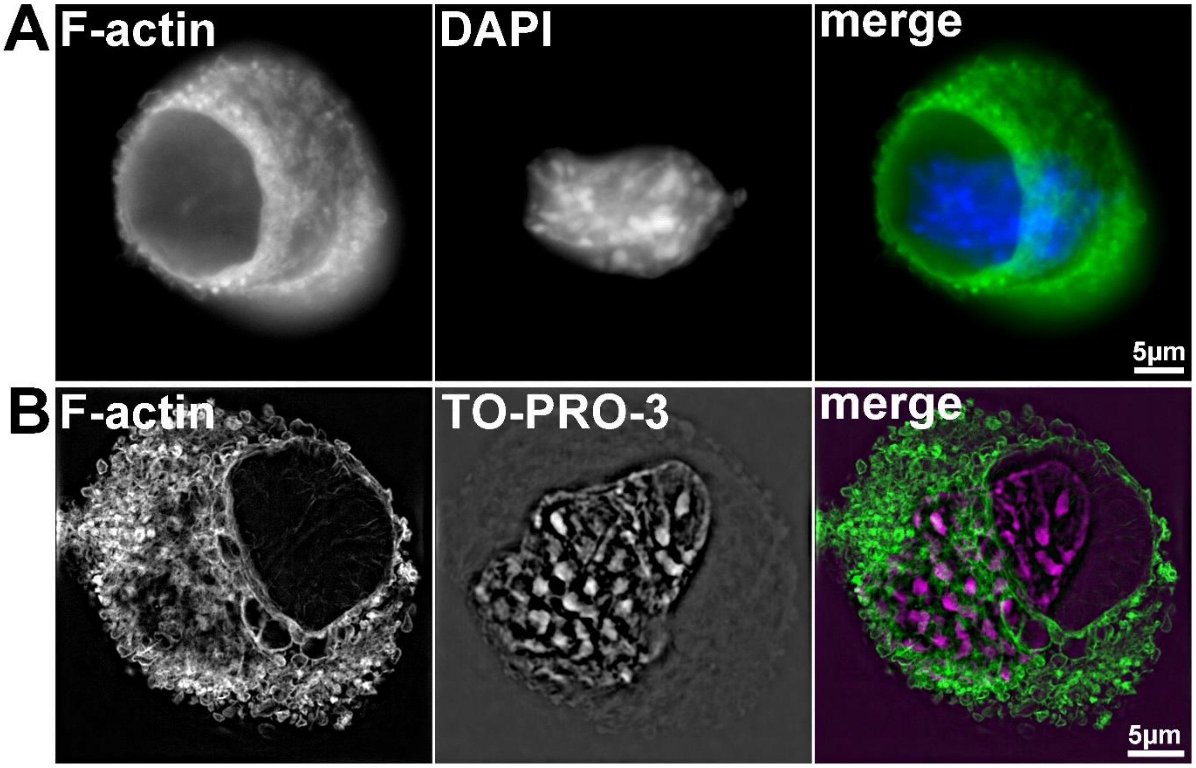

Morphology of the actin cytoskeleton in spreading cells simultaneously suppressed for Arp3 and FMNL2/3 expression. (A) Widefield fluorescence and **(B)** SIM imaging of Arp3 and FMNL2/3-depleted, spreading cells stained with phalloidin (F-actin) and the nucleus, as indicated. Note the formation of an adhesion area of reduced F-actin intensity, which did not simply coincide with the position of the nucleus.

Figure S15.

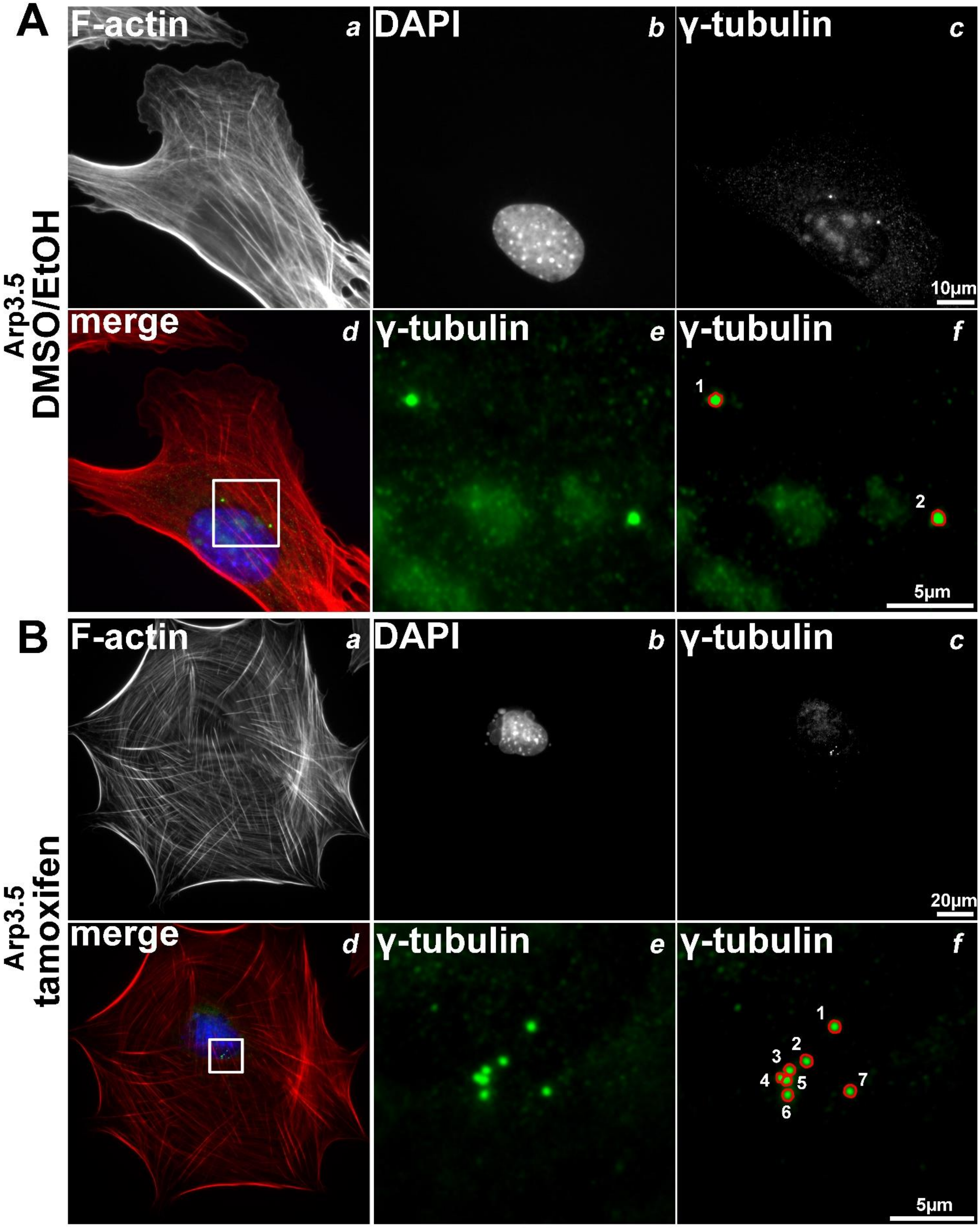
A
F-actin
a
DAPI
b
γ-tubulin
c
10µm
merge
d
γ-tubulin
e
γ-tubulin
f
1
2
5µm
Arp3.5
DMSO/EtOH
B
F-actin
a
DAPI
b
γ-tubulin
c
20µm
merge
d
γ-tubulin
e
γ-tubulin
f
1
2
3
4
5
6
7
5µm
Arp3.5
tamoxifen

Arp3 depletion causes an increase of centrosome and nucleus numbers as well as nuclear deformation, clone Arp3.5. Representative raw data for clone Arp3.5 corresponding to quantifications shown in Fig. 10C-E.

Figure S16.

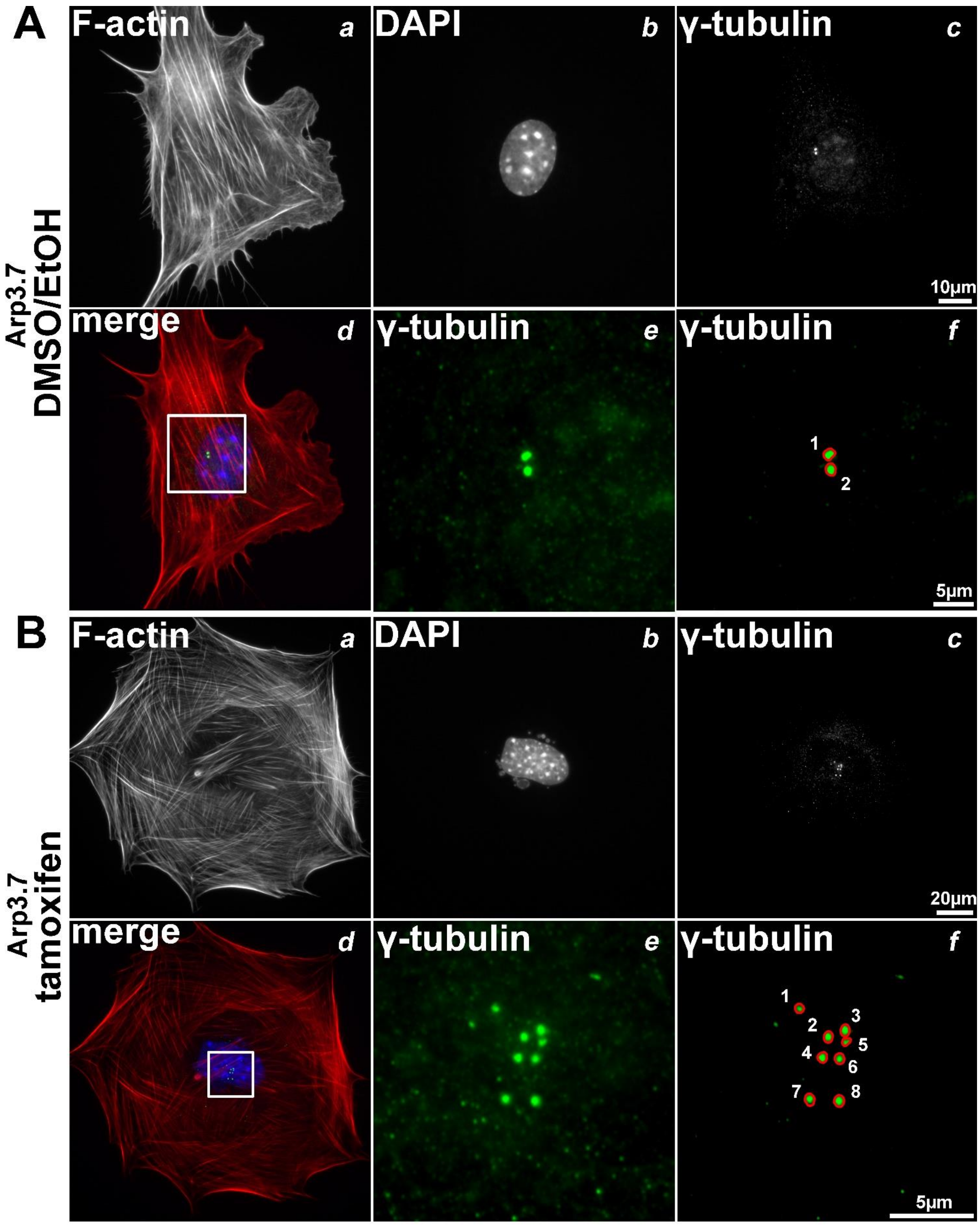
A
F-actin
a
DAPI
b
γ-tubulin
c
10μm
merge
d
γ-tubulin
e
γ-tubulin
f
1
2
5μm
Arp3.7
DMSO/EtOH
B
F-actin
a
DAPI
b
γ-tubulin
c
20μm
merge
d
γ-tubulin
e
γ-tubulin
f
1
2
3
4
5
6
7
8
5μm
Arp3.7
tamoxifen

Arp3 depletion causes an increase of centrosome and nucleus numbers as well as nuclear deformation, clone Arp3.7. Representative raw data for clone Arp3.7 corresponding to quantifications shown in Fig. 10C-E.

Supplementary Videos

Video S1. Arp2/3 complex knockout fibroblasts efficiently spread using numerous filopodia. Time-lapse microscopy of *Actr3*$^{fl/fl}$ MEFs (clone Arp3.7) with (right) or without (left) tamoxifen treatment, and allowed to spread on fibronectin. Time is given in minutes and seconds.

Video S2. Wound healing migration with and without acute reduction of Arp2/3 complex function. Time-lapse microscopy of *Actr3*$^{fl/fl}$ MEFs (clone Arp3.5) with (right) or without (left) tamoxifen treatment, and stimulated to migrate upon cell monolayer wounding. Time is given in hours and minutes (bottom right of each panel), and bars at the bottom left correspond to 50µm.

Video S3. 3D SIM projection of phalloidin-stained control cell. DMSO/EtOH-treated *Actr3*$^{fl/fl}$ cell (clone Arp3.19) transfected with mock-RNAi vector and allowed to spread for 15 minutes.

Video S4. 3D SIM projection of phalloidin-stained Arp3/FMNL2/3-depleted cell. Tamoxifen-treated *Actr3*$^{fl/fl}$ cell (clone Arp3.19) transfected with FMNL2+3-RNAi vectors and allowed to spread for 15 minutes. Cells suppressed for both Arp2/3 complex and FMNL2/3 formins appear to adopt a highly peculiar shape (thick flattened disks), likely caused by actin assembly deficiencies at the cell periphery caused by this treatment.

Video S5. 3D SIM projection of phalloidin-stained Arp3/FMNL2/3-depleted cell, representative example 2. Tamoxifen-treated *Actr3*$^{fl/fl}$ cell (clone Arp3.19) transfected with FMNL2+3-RNAi vectors and allowed to spread for 15 minutes. Note that in this case, the optical sectioning allows a view from inside the cell, revealing the typical F-actin-deficient area (see also legend to Fig. S14) to be positioned at the ventral cell surface.

Video S6. 3D SIM projection of Arp3/FMNL2/3-depleted cell co-stained with phalloidin (green) and the nucleus with TO-PRO-3 (pink). Tamoxifen-treated *Actr3*$^{fl/fl}$ cell (clone Arp3.19) transfected with FMNL2+3-RNAi vectors and allowed to spread for 15 minutes. Again, the F-actin-deficient zone at the ventral cell surface does not fully co-inside with the position of the nucleus.
